# Dysregulation of endothelial colony-forming cell function by a negative feedback loop of circulating miR-146a and -146b in cardiovascular disease patients

**DOI:** 10.1371/journal.pone.0181562

**Published:** 2017-07-20

**Authors:** Ting-Yu Chang, Wei-Chi Tsai, Tse-Shun Huang, Shu-Han Su, Chih-Young Chang, Hsiu-Yen Ma, Chun-Hsien Wu, Chih-Yung Yang, Chi-Hung Lin, Po-Hsun Huang, Cheng-Chung Cheng, Shu-Meng Cheng, Hsei-Wei Wang

**Affiliations:** 1 Research Center of Translational Medicine, Taipei Medical University, Taipei, Taiwan; 2 Division of Cardiology, Department of Internal Medicine, Tri-Service General Hospital, National Defense Medical Center, Taipei, Taiwan; 3 Institute of Microbiology and Immunology, National Yang-Ming University, Taipei, Taiwan; 4 Institute of Engineering in Medicine, University of California, San Diego, United States of America; 5 Cardiovascular Research Center, National Yang-Ming University, Taipei, Taiwan; 6 Division of Cardiology, Department of Medicine, Taipei Veterans General Hospital and Institute of Clinical Medicine, Taipei, Taiwan; Centro Cardiologico Monzino, ITALY

## Abstract

Functional impairment of endothelial colony-forming cells (ECFCs), a specific cell lineage of endothelial progenitor cells (EPCs) is highly associated with the severity of coronary artery disease (CAD), the most common type of cardiovascular disease (CVD). Emerging evidence show that circulating microRNAs (miRNAs) in CAD patients’ body fluid hold a great potential as biomarkers. However, our knowledge of the role of circulating miRNA in regulating the function of ECFCs and the progression of CAD is still in its infancy. We showed that when ECFCs from healthy volunteers were incubated with conditioned medium or purified exosomes of cultured CAD ECFCs, the secretory factors from CAD ECFCs dysregulated migration and tube formation ability of healthy ECFCs. It is known that exosomes influence the physiology of recipient cells by introducing RNAs including miRNAs. By using small RNA sequencing (smRNA-seq), we deciphered the circulating miRNome in the plasma of healthy individual and CAD patients, and found that the plasma miRNA spectrum from CAD patients was significantly different from that of healthy control. Interestingly, smRNA-seq of both healthy and CAD ECFCs showed that twelve miRNAs that had a higher expression in the plasma of CAD patients also showed higher expression in CAD ECFCs when compared with healthy control. This result suggests that these miRNAs may be involved in the regulation of ECFC functions. For identification of potential mRNA targets of the differentially expressed miRNA in CAD patients, cDNA microarray analysis was performed to identify the angiogenesis-related genes that were down-regulated in CAD ECFCs and Pearson’s correlation were used to identify miRNAs that were negatively correlated with the identified angiogenesis-related genes. RT-qPCR analysis of the five miRNAs that negatively correlated with the down-regulated angiogenesis-related genes in plasma and ECFC of CAD patients showed miR-146a-5p and miR-146b-5p up-regulation compared to healthy control. Knockdown of miR-146a-5p or miR-146b-5p in CAD ECFCs enhanced migration and tube formation activity in diseased ECFCs. Contrarily, overexpression of miR-146a-5p or miR-146b-5p in healthy ECFC repressed migration and tube formation in ECFCs. TargetScan analysis showed that miR-146a-5p and miR-146b-5p target many of the angiogenesis-related genes that were down-regulated in CAD ECFCs. Knockdown of miR-146a-5p or miR-146b-5p restores CAV1 and RHOJ levels in CAD ECFCs. Reporter assays confirmed the direct binding and repression of miR-146a-5p and miR-146b-5p to the 3’-UTR of mRNA of RHOJ, a positive regulator of angiogenic potential in endothelial cells. Consistently, RHOJ knockdown inhibited the migration and tube formation ability in ECFCs. Collectively, we discovered the dysregulation of miR-146a-5p/RHOJ and miR-146b-5p/RHOJ axis in the plasma and ECFCs of CAD patients that could be used as biomarkers or therapeutic targets for CAD and other angiogenesis-related diseases.

## Introduction

Coronary artery disease (CAD), the most common type of heart disease and the single most common leading cause of death in developed countries, starts with a progressive impairment of endothelial integrity and function [1, 2]. Emerging evidence suggests that refurbishment of damaged microcirculation is not solely the result of proliferation of local endothelial cells, but also largely depends on bone marrow-derived endothelial progenitor cells (EPCs). EPCs participate in vessel homeostasis (reviewed in [3]). A negative correlation between the number of EPCs in peripheral blood with angiogenesis-related phenotype [4] and severity of CAD [5] had been demonstrated. In addition to the circulating EPCs count, the regulation of adult angiogenesis and vasculogenesis are also highly dependent on the migratory activity of EPCs, and dysfunctional EPCs are observed to contribute to CAD pathogenesis [6, 7]. EPCs are therefore considered as biomarker of vascular health, prognosis factor of CAD, and as a potential therapeutic agent in cardiovascular diseases.

Since the first identification of EPCs in 1997 [8], EPCs have been extensively studied for their ability to repair vessels [9]. Transplanted EPCs show capability of direct incorporation into the vasculature for recovery of ischemia [10, 11]. EPCs can be divided into two distinct groups according to phenotypic lineages, known as the haematopoietic (early EPCs or myeloid angiogenic cells [MACs]) and endothelial lineage (late EPCs or endothelial colony forming cells [ECFCs]) [12]. MACs do not give rise to endothelial cells, however, it can promote angiogenesis through paracrine mechanism [13]. ECFCs can be obtained from mononuclear cells (MNCs) in peripheral blood and are identified using specific cell surface markers, including hematopoietic stem cell marker CD34, endothelial lineage surface antigen CD31 (also known as platelet/endothelial cell adhesion molecule 1, PECAM1), VE-cadherin, and vascular endothelial growth factor receptor 2/kinase insert domain receptor (VEGFR2/KDR) [14–18]. Accumulating evidence indicate that different mechanisms are responsible for reducing the amount and activities of ECFCs. For example, decreased expression of angiogenic cytokine CXCL12, also named as stromal cell-derived factor 1 (SDF1), resulted in apoptosis of ECFCs [19, 20]. Reduced levels of VEGF, a known mitogen for ECFC, suppressed proliferation and activation of circulating EPCs and overexpression of VEGF augmented ECFCs proliferation *in vitro* and neovascularization *in vivo* [21].

In addition to regulating protein-coding genes in EPCs, emerging evidence shows that the expression of various microRNAs (miRNAs) is also important for the function of EPCs and consequently in the pathogenesis of CAD. In our previous report, we detected and compared the levels of miRNA in peripheral blood and umbilical cord blood ECFCs, we found that the ECFCs show high and low vasculogenic ability, respectively. We also showed that miR-31 was more abundant in umbilical cord blood-derived ECFCs by small RNA sequencing (smRNA-seq) [22]. Transfer of miR-31 to peripheral blood-derived ECFCs increased cell migration and microtubule formation functions in ECFCs [22]. On the other hand, anti-angiogenic miR-221 and miR-222, which modulate the angiogenic properties of human umbilical vein endothelial cells through targeting c-kit and NO synthase [23], were significantly up-regulated in patients with CADs [24, 25]. This data illustrates the critical role of miRNAs in ECFC biological functions. In addition to tissue RNAs, circulating miRNAs in body fluids have emerged as potential new biomarkers for diseases [26–28] and novel therapeutic targets [29, 30].

Expression of many miRNAs is associated with the severity of CAD [31, 32]. Recently, many reports including ours showed the negative correlation between the levels of ECFCs and circulating miRNAs with the expression of VEGF [33–35] or its receptor VEGFR [36, 37] in CAD patients. These data indicate that secretory miRNAs from diseased ECFCs might diminish the functionality of global healthy ECFCs that consequently leads to pathogenesis of artery disease. Identifying the secretory factors may help to block the “negative loop” from generating defective ECFCs. The mechanisms of angiogenesis, besides mitogenic VEGF signaling, remain largely unknown. Here, by using smRNA-seq, we deciphered the miRNome pattern of CAD ECFCs and plasma, and identified miR-146a-5p and miR-146b-5p as potential angiogenic regulatory miRNAs that target an endothelial cell-restricted Rho GTPase, RHOJ. Knockdown of miR-146a-5p or miR-146b-5p enhanced migration and tube formation activity in CAD ECFCs. Consistently, overexpression of miR-146a-5p or miR-146b-5p in healthy ECFCs repressed ECFCs functionality. Collectively, these finding provide evidence of dysregulation of miR-146a-5p/RHOJ and miR-146b-5p/RHOJ axis in CAD pathogenesis and supports its potential use as an effective prognostic and therapeutic implications in CAD.

## Materials and methods

### Ethics statement

This study was approved by the institutional Ethics Committee Review Boards of Tri-Service General Hospital and the Taipei Veteran General Hospital (IRB#: 1-103-05-061). Written informed consents were obtained from all participants. The protocols of this study were consistent with the ethical guidelines of 1975 Helsinki Declaration.

### Isolation and culture of ECFCs

ECFCs were isolated from peripheral blood samples of HLTY individuals and CAD patients and characterized by flow cytometry (FACS) analysis using hematopoietic stem cell and endothelial lineage surface markers as previously described [36]. CAD patients who were diagnosed by using Cardiac Catheterization were considered as patients with CAD and included in this study. Mononuclear cells (MNCs) were isolated using Histopaque-1077 (1.077 g/ml, Sigma, St. Louis, MO, USA) by centrifugation. The MNCs isolated were resuspended in endothelial growth medium-2 with complete supplements (Lonza Ltd, Basel, Switzerland) and seeded onto plates coated with fibronectin. After 14–21 days cultivation at 37°C with 5% CO_2_, ECFCs with typical phenotype of cobblestone-shaped and monolayer growth pattern were obtained. All the ECFCs used for functional analysis and RNA extraction were cultured within six passages.

### RNA extraction and reverse transcription and quantitative PCR (RT-qPCR)

Total RNA was extracted from ECFCs using TRIzol reagent (Invitrogen, Carlsbad, CA, USA) and plasma using TRIzol LS reagent (Invitrogen) following the manufacturer’s protocol. For miRNA detection, 150 ng of total RNA was used for RT using Universal cDNA Synthesis and SYBR^®^ Green Master Mix kits (EXIQON, Vedbaek, Denmark). Real-time PCR reactions were performed with miRCURY LNA^TM^ Universal RT microRNA PCR LNA^TM^ PCR primer set (EXIQON) using CFX connect^TM^ real-time PCR detection system (Bio-Rad, Hercules, CA, USA) in Bio-Rad CFX96 Real-Time PCR Detection System. The expression of miRNAs was normalized to U6 small nuclear RNA. For mRNA detection, 2 μg of total RNA was used for RT with Oligo(dT)_12-18_ using SuperScript^III^ RT (Invitrogen). The expression of each gene was quantified by iQ^TM^ SYBR Green Supermix (BioRad) using CFX96 Real-Time PCR Detection System and normalized to GAPDH.

### smRNA-seq and data analysis

Total RNAs purified from plasma and ECFCs of HLTY individuals and CAD patients were sequenced using Illumina MiSeq (Illumina, San Diego, CA, USA) following the standard manufacturer’s procedure. Sequencing results were analyzed using an in-house bioinformatics pipeline [38]. Expression levels of miRNAs were calculated as read per millions of mapped reads (RPM). The p-value and false discovery rate of differential miRNA expression were processed by DESeq2 [39]. TargetScan 7.0 (www.targetscan.org) was used to predict target genes and binding sites of miR-146a-5p and miR-146b-5p.

### Cloning miRNAs and expression of miRNAs and anti-miRNAs

Genomic DNA fragments containing precursor sequence of miR-146a-5p and miR-146b-5p was cloned into pL4-V5-CPO-B, a lentiviral-based expression vector using miR-146a-5p primer pairs: forward: 5’-ATAGGATCCATCATGCATGGCTCATTTTT-3’; reverse: 5’-ATAGGATCCAGCTACTTGGAACCCTGCTT-3’; and miR-146b-5p primer pairs: forward: 5’-ATAGGATCCAGACCCTCCCTGGAATAGGA-3’; reverse: 5’-ATAGGATCCGAGCACTGAGGAAGGACCAG-3’. miRZip^TM^ lentivector-based anti-miRNAs expresses anti-miRNA-146a-5p (5’-GTGAGAACGGAATTCCATGGATTCTTCCTGTCAGAACCCATGGAATTCAGTTCTCATTTTT-3’) and anti-miR-146b-5p (5’-GGATCCGTGAGAACCGAATTCCATCGGATCTTCCTGTCAGAGCCTATGGAATTCAGTTCTCATTTTTGAATTC-3’) was purchased from System Biosciences (Mountain View, CA, USA). Lentiviruses expressing miRNAs and anti-miRNAs were produced in 293T cells and used for transduction.

### Transwell cell migration and tube formation assays

For migration assay, 600 μl of EGM2 medium supplemented with 10% FBS were added to the lower chamber, while 5x10^4^ ECFCs in 100 μl of EGM2 medium were seeded on the upper chamber with 8 μm pore size of Costar Transwell Polycarbonate Permeable Supports (Corning, Corning, New York, USA). Six hours after incubation at 37°C with 5% CO_2_, suspension cells were removed and the membranes were fixed with 4% paraformaldehyde for 15 minutes at room temperature. The migrated cells were then stained with Hoechst 33342 reagents (Sigma) for 30 minutes and counted under fluorescent microscope in five representative fields. The images were quantified using MetaMorph (Molecular Devices, Sunnyvale, CA, USA).

For *in vitro* tube formation assay, 96-well plate were coated with Basement Membrane Extract (BME, 3433-005-01, Trevigen Inc., Gaithersburg, MD, USA) (50 μl per well) at 37°C for an hour as described previously [33]. 1x10^4^ ECFCs in 100μl medium were placed onto the matrix and incubated at 37°C for 3 hours. The tube structures were visualized under an inverted light microscope (100 X). Five microscopic fields were captured in each group and total tube length was calculated using MetaMorph (Molecular Devices). All data were obtained from three independent experiments with triplication.

### Luciferase reporter assays

Luciferase reporter plasmids were constructed by cloning the predicted miRNA-binding site on 3’-UTR of CAV1 and RHOJ into pGL3-Basic plasmid (Promega, Madison, WI, USA) using specific primers for CAV1, forward: 5’-ATTACTAGTGCAACTCGCTTTAGGTCAGC-3’; reverse: 5’-ATTAAGCTTGGCCATGTGTCACAGCATAA-3’ and RHOJ, forward: 5’-ATTGAGCTCTTTTCCATGTATTGCCACGA-3’; forward: 5’-ATTAAGCTTTTACATCATTGGGCACAGGA-3’. The luciferase reporter plasmids containing mutations on miR-146a-5p and miR-146b-5p binding sites in the 3’UTR of RHOJ was created using QuikChange Site-Directed Mutagenesis Kit (Stratagene, San Diego, CA, USA). Reporter assays were performed by transiently co-transfecting 293T cells with reporter plasmids and vector overexpressing miR-146a-5p or miR-146b-5p using Lipofectamine 2000 reagent (Life Technologies). Renilla luciferase was used to normalize transfection efficiency. Forty-eight hours after transfection, total cell lysates were measured for the luciferase activities by the Dual-Luciferase Reporter Assay System (Promega), according to the manufacturer's instruction.

### Statistical analyses

Statistical analysis of gene and miRNA expression measured by qPCR was performed using *Student’s-t* test and indicated as n.s: not significant; *p<0.05; **p<0.01; ***p<0.001.

## Results

### Conditioned medium (CM) derived from coronary artery disease (CAD) ECFCs inhibited migration and tube formation ability of healthy (HLTY) ECFCs

**Fig 1:**
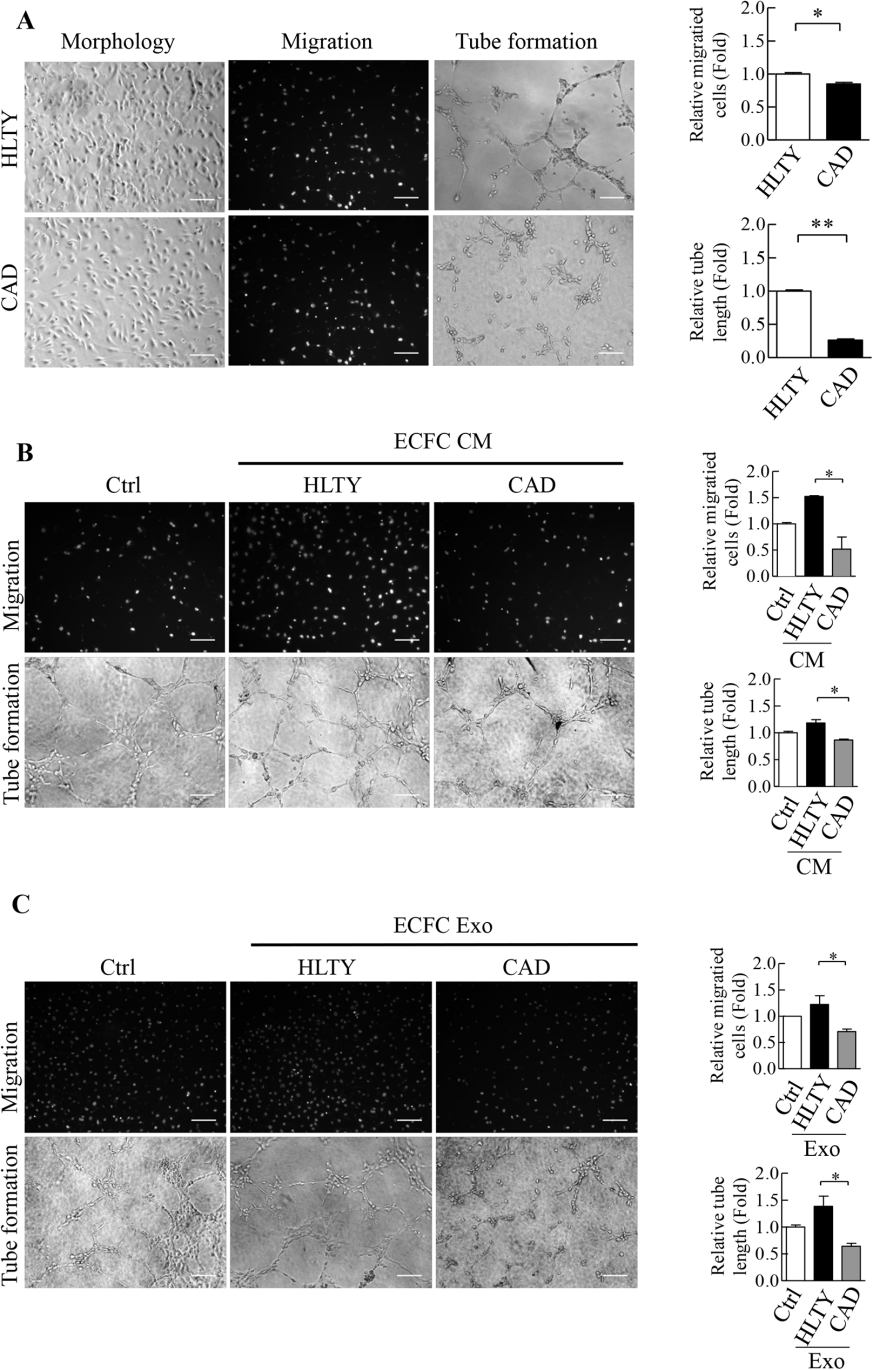
Reduced angiogenic activities of ECFCs from CAD patients. (A) Morphology of CAD and HLTY ECFCs (Left panel). ECFCs were subjected to transwell cell migration and Matrigel tube formation assays. Migrated cells were photographed (representative picture are shown in middle left panel) and quantitated (right upper panel, n = 3). Tube lengths of formed microvascular structure (representative pictures are shown in middle right panel) were measured (right upper panel, n = 3). *p<0.05, **p<0.01 by *Student’s-t* test. (B) Representative image (left panel) and quantification (left panel) of migration and tube formation of HLTY ECFCs treated with conditioned medium from CAD and HLTY ECFCs. (C) Representative image (left panel) and quantification (right panel) of migration and tube formation of HLTY ECFCs treated with exosomes purified from culture supernatants of CAD and HLTY ECFCs.

Cobblestone-like ECFCs obtained from the peripheral blood (PB) of HLTY donor and CAD patients were first characterized by fluorescence-activated cell sorter (FACS) as described [22]. Both ECFCs were confirmed negative for hematopoietic marker CD45 and positive for endothelial markers CD34, KDR, CD31 and VE-cadherin (S1 Fig). Consistent with previous findings [33], the migratory and the tube formation activity of CAD ECFCs were less efficient than HLTY ECFCs (Fig 1A). Interestingly, in this study we found that CM derived from CAD ECFCs significantly reduced the migration and tube formation abilities of HLTY ECFCs (Fig 1B). Emerging evidence showed that circulating exosomes function as intercellular transporter delivering cargos to recipient cells [40]. To determine if CAD ECFCs derived exosomes are responsible for CM-mediated inhibition of cell migration and tube formation abilities, we isolated exosomes from CM derived from CAD ECFCS and used them to treat HLTY ECFCs. As expected, exosomes derived from CAD ECFCs inhibited the migration and tube formation abilities of HLTY ECFCs (Fig 1C). These data indicate that secretory factors from CAD ECFCs might diminish the functionality of HLTY ECFCs. Identifying the secretory factors may help to block the “negative loop” for generating defective ECFCs.

### Analysis of miRNA expression profile in plasma and ECFCs of HLTY individuals and CAD patients

**Fig 2:**
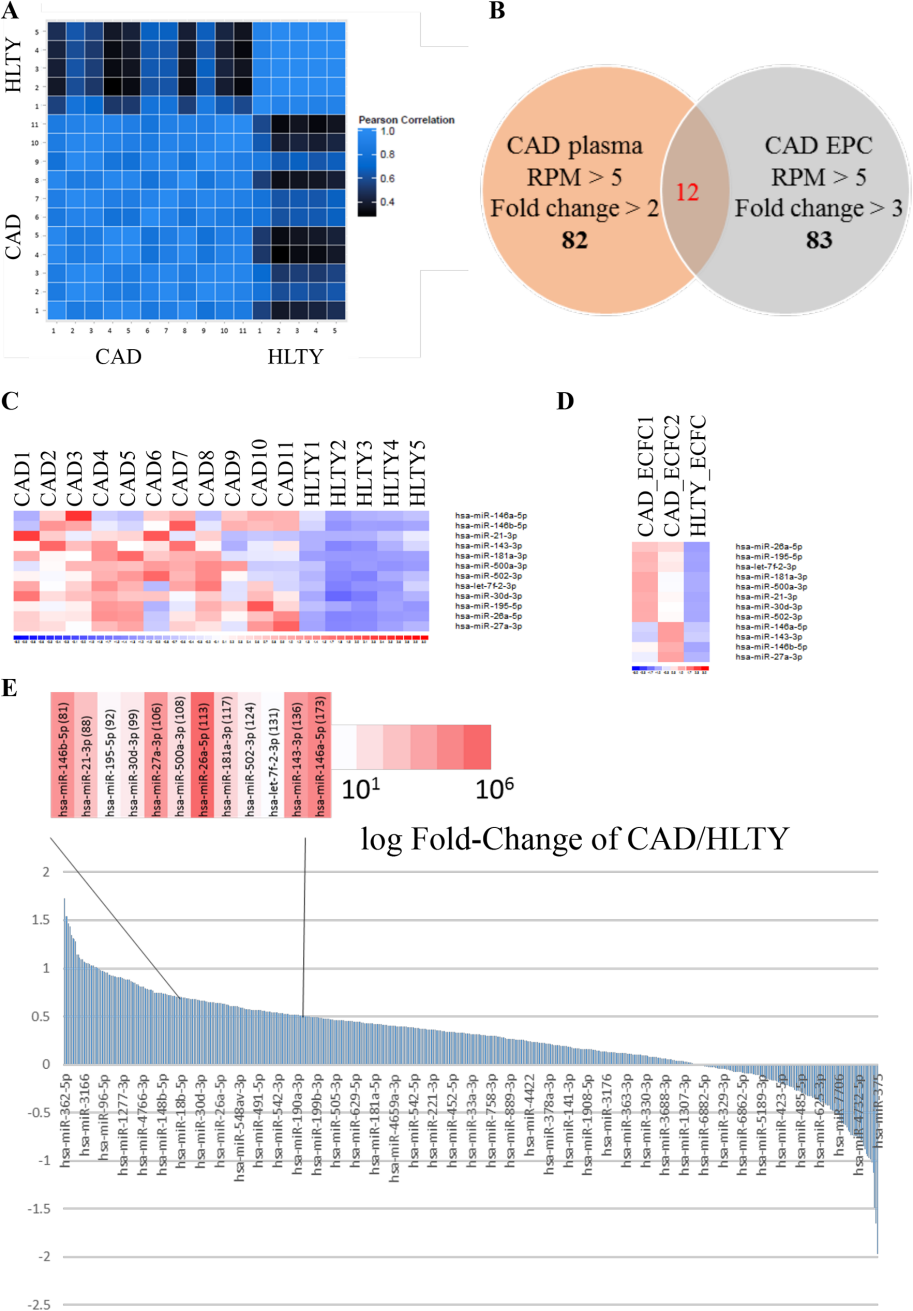
smRNA-seq data analysis. (A) Pairwise expression correlation of miRNAs profiles in plasma from CAD patients and HLTY individuals. Significant difference was observed between the two groups. (B) Venn diagram of miRNAs highly expressed in plasma and ECFCs and significantly up-regulated in patients with CAD. (C and D) Heat map of the expression profile of the 12 miRNAs that were most highly expressed in CAD plasma (C) and ECFCs (D) of CAD patients and HLTY individuals. (E) Histogram demonstrating the expression pattern of plasma miRNAs. Expression level was presented as log10 (y-axis). The 12 miRNAs shown in (D) are highlighted in red (upper panel) corresponding to their average expression level (read per millions of mapped reads, RPM). The numbers in red brackets represent the rank sorted by fold change (CAD/HLTY).

Circulating miRNAs have been found in exosomes [41]. In addition, miRNAs are also known to be actively or passively released in circulation as intercellular communication molecules [42, 43]. To determine whether circulating miRNAs play a role in regulating the physiological function of ECFCs in PB, total RNA harvested from plasma of both HLTY individuals and CAD patients were purified and used for smRNA-seq. Hemolysis was monitored by OD_480_ to avoid background miRNA signals from red blood cells. A pilot study of exosome miRNAs sequencing confirmed similar miRNA signature of exosome and plasma miRNAs. To simplify the processing step, we aimed to purify the miRNA from plasma. Total RNA from plasma of 5 individuals were pooled for preparation of a library for smRNA-seq. About 4 to 5 million of raw reads were obtained using Illumina MiSeq. Total reads were first trimmed for adapter sequence and filtered with read length. Reads with more than 90% of the bases that reached a score of ≤Q30 (Phred score) were preserved for further analysis. Unique read counts and length distribution peaks were summarized (Table 1). Reads were then mapped to known classes of small RNAs, including tRNA, rRNA, snoRNA, snRNA, and miRNAs. The expression pattern of miRNAs was measured by pair-wise Pearson correlation coefficient and demonstrated by heat map (Fig 2A). The result indicates the differences between the miRNA expression profiles of CAD and HLTY specimens (Fig 2A).

**Table 1:** Summary of unique read counts and length distribution peaks.

	Total reads	Trimmed read counts	Filtered read counts	Qualified read counts	Unique read counts	Length distribution peaks
CAD1	4,651,116	4,536,013	2,960,392	2,774,394	53,648	22
CAD2	4,206,598	3,881,419	2,854,887	2,697,120	55,860	22
CAD3	2,655,421	2,564,003	1,865,169	1,750,926	32,112	22
CAD4	3,897,204	3,776,896	2,013,601	1,885,080	35,129	22
CAD5	4,728,743	4,573,117	2,864,443	2,666,032	48,953	22
CAD6	4,198,927	4,130,925	2,025,038	1,886,691	42,670	22
CAD7	4,050,474	3,922,128	1,961,507	1,841,568	38,073	22
CAD8	4,617,782	4,534,474	2,055,919	1,891,884	36,983	22
CAD10	4,126,428	4,037,220	2,173,824	1,973,659	30,864	22
CAD13	4,835,962	4,705,894	2,945,331	2,784,195	48,382	22
CAD14	4,385,133	2,457,221	2,034,991	1,936,697	22,692	23
CAD15	5,185,804	4,926,514	4,216,796	4,021,940	51,845	22
CAD16	3,434,029	3,175,552	2,269,138	2,156,805	38,823	22
CAD17	2,653,254	2,446,162	2,002,795	1,903,490	37,623	22
CAD18	4,059,163	3,339,940	2,604,293	2,454,509	75,334	22
HLTY1	3,070,911	2,942,588	2,242,970	2,109,891	40,060	22
HLTY2	3,953,752	3,839,484	1,591,844	1,496,697	38,176	22
HLTY3	3,064,746	2,223,058	1,352,087	1,280,965	27,188	23
HLTY4	4,516,803	4,318,367	3,708,552	3,446,998	45,169	22
HLTY5	3,684,820	3,592,341	3,190,895	2,991,052	36,175	27
HLTY6	4,621,155	4,464,923	2,053,745	1,864,866	31,731	22
HLTY7	5,198,374	4,961,039	3,988,350	3,762,464	40,641	22
HLTY8	6,784,266	6,674,934	3,728,209	3,549,060	70,502	22
HLTY9	3,902,506	3,743,951	2,466,278	2,326,669	40,674	22
HLTY10	4,830,544	4,684,389	3,643,901	3,307,187	47,401	22
HLTY11	3,523,753	2,958,091	1,180,215	1,108,697	40,235	22
HLTY12	4,688,801	2,348,685	1,153,032	1,083,961	29,928	22
HLTY13	2,892,615	2,677,872	642,667	606,574	15,784	22
HLTY14	3,803,727	3,556,221	1,030,545	972,294	33,144	22
HLTY15	4,968,729	4,848,883	4,162,616	3,897,751	51,455	22

To identify miRNA candidates that are dysregulated in CAD ECFC, we compared the miRNA expression profile of CAD plasma and ECFC to that of HLTY subjects. We found 82 and 83 miRNAs were up-regulated in CAD plasma and ECFC versus HLTY control, respectively (Fig 2B). Heat maps of these miRNAs in plasma and ECFC of HLTY individuals and CAD patients are shown (S2A and S2B Fig, respectively). Among them, 12 miRNAs were up-regulated in both CAD plasma and ECFC (Fig 2B). Heat map showed significant differences of these 12 miRNAs in CAD plasma and ECFC versus HLTY control (Fig 2C and 2D, respectively). When the plasma miRNAs that were dysregulated in CAD patients were plotted by fold-change (log_10_), the 12 miRNAs that were up-regulated in both CAD plasma and ECFC were ranked among the top 50% of highly unregulated plasma miRNAs, as highlighted on top of Fig 2E. The red color depth represents the average level of miRNAs (read per millions of mapped reads, RPM) in plasma of CAD patients. The 12 highly expressed miRNAs in CAD plasma and ECFC are listed in Table 2.

**Table 2:** CAD plasma and EPC up-regulated miRNAs ranked by CAD plasma expression profile.

No.	Name	Plasma					EPC		
		CAD	HLTY	Fold change	p-value	FDR	CAD	HLTY	Fold change
**1**	**hsa-miR-26a-5p**	**168803.2**	**67479.1**	**2.5**	**1.47E-08**	**2.34E-07**	**27959.5**	**2127.5**	**13.1**
**2**	**hsa-miR-146a-5p**	**36837.9**	**17721.6**	**2.1**	**0.03833**	**0.09911**	**7487.1**	**970.9**	**7.7**
**3**	**hsa-miR-146b-5p**	**16263.3**	**4634**	**3.5**	**8.38E-05**	**0.00055**	**1543.1**	**42.4**	**36.4**
**4**	**hsa-miR-27a-3p**	**12003.4**	**4428.2**	**2.7**	**2.92E-06**	**2.61E-05**	**37027.4**	**1894.1**	**19.5**
**5**	**hsa-miR-143-3p**	**9492.2**	**4098**	**2.3**	**0.00256**	**0.01046**	**1183.6**	**69**	**17.2**
6	hsa-miR-21-3p	945.1	355.8	2.7	0.01109	0.036	13785.4	10.6	1300.5
7	hsa-miR-181a-3p	326.9	145.9	2.2	3.69E-07	3.99E-06	1818.7	84.9	21.4
8	hsa-miR-500a-3p	205.2	82.7	2.5	1.96E-05	0.00014	364.3	5.3	68.7
9	hsa-miR-30d-3p	107.6	35.5	3	3.25E-07	3.62E-06	86	5.3	16.2
10	hsa-miR-502-3p	66.5	29.3	2.3	0.00023	0.00134	208.6	5.3	39.4
11	hsa-miR-195-5p	56	18.1	3.1	0.00115	0.00535	274.5	31.8	8.6
12	hsa-let-7f-2-3p	34.2	13.7	2.5	0.00509	0.01919	52.5	5.3	9.9

### Identify miRNAs with potential of targeting angiogenesis-related genes

**Fig 3:**
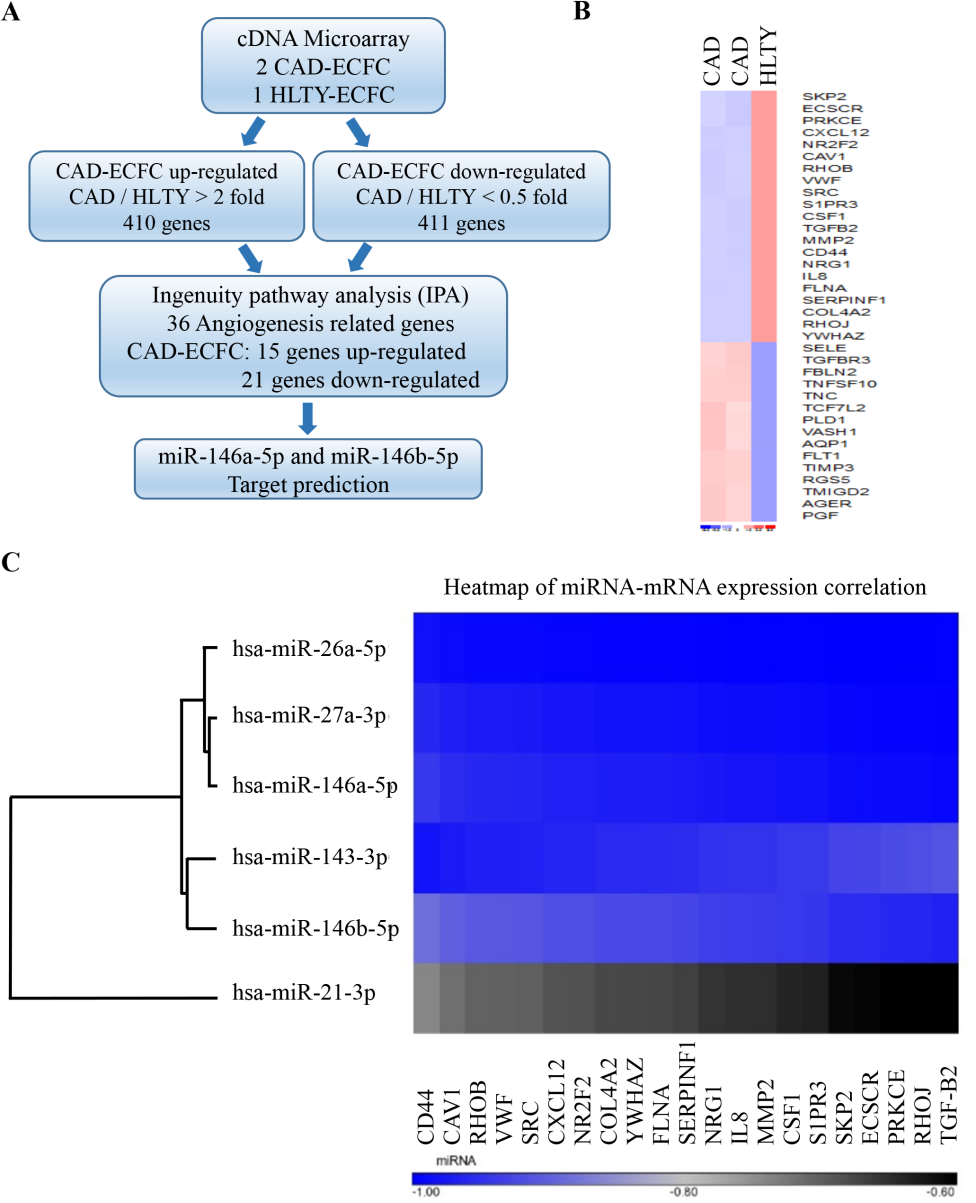
Combined cDNA Microarray and smRNA-seq analysis reveal correlation between down-regulated angiogenesis-related genes and up-regulated circulating miRNAs. (A) Flowchart of the analysis pipeline. The mRNA expression profile in CAD and HLTY ECFCs carried out by cDNA microarray. 401 and 411 transcripts with 2-fold up- or down-regulation, respectively, in CAD ECFCs compared with healthy control were identified. Functional classification using Ingenuity Pathway Analysis (IPA) revealed 36 angiogenesis-related genes. TargetScan was carried out to predict potential miR-146a-5p and miR-146b-5p binding sites on the 36 identified angiogenesis-related genes. (B) Heat map of gene expression profile of 36 angiogenesis-related genes in CAD and HLTY ECFCs. (C) Heat map of expression correlation of top 6 up-regulated miRNAs in CAD plasma and 21 angiogenesis-related genes down-regulated in CAD ECFCs. Pearson correlation coefficient was calculated to demonstrate the degree of negative correlation. The top first 5 miRNAs show significant negative correlation with the 21 angiogenesis-related genes down-regulated in CAD ECFCs.

We hypothesized that miRNAs up-regulated in CAD ECFCs might be secreted in plasma and uptake by HLTY ECFCs, thereby dysregulating mRNAs expression and functionality of HLTY ECFCs and making them more CAD ECFCs-like. Therefore, we performed cDNA microarray analysis to identify genes that were up- or down-regulated in CAD ECFCs. By using RNA prepared from ECFCs isolated from two CAD patients and one HLTY individual, we identified 410 and 411 genes that were at least 2-fold up- and down-regulated in CAD ECFCs, respectively (Fig 3A). Following molecular function classification and network analysis of those up- and down-regulated genes using Ingenuity Pathway Analysis™ (IPA), 36 angiogenesis-related genes with 15 and 21 genes up- and down-regulated in CAD ECFCs were identified (Fig 3A). Heat map showed significant differences between the 36 mRNAs in CAD ECFC versus HLTY control (Fig 3B). We hypothesized that miRNAs that are up-regulated in CAD plasma may be involved in dysregulating the expression of those angiogenesis-related genes. Therefore, a negative expression correlation between miRNAs up-regulated in CAD plasma and the angiogenesis-related genes were analyzed using Pearson’s correlation coefficient. Out of the top 6 miRNAs that are highly expressed in CAD plasma shown in Table 2, we observed a significant negative correlation between the top 5 highly expressed miRNAs and the 21 angiogenesis-related genes that are down-regulated in CAD ECFCs (Fig 3C). Therefore, those 5 miRNAs were chosen for further analysis.

### Increased miR-146a-5p and miR-146b-5p levels in the plasma and ECFCs of patients with CAD

**Fig 4:**
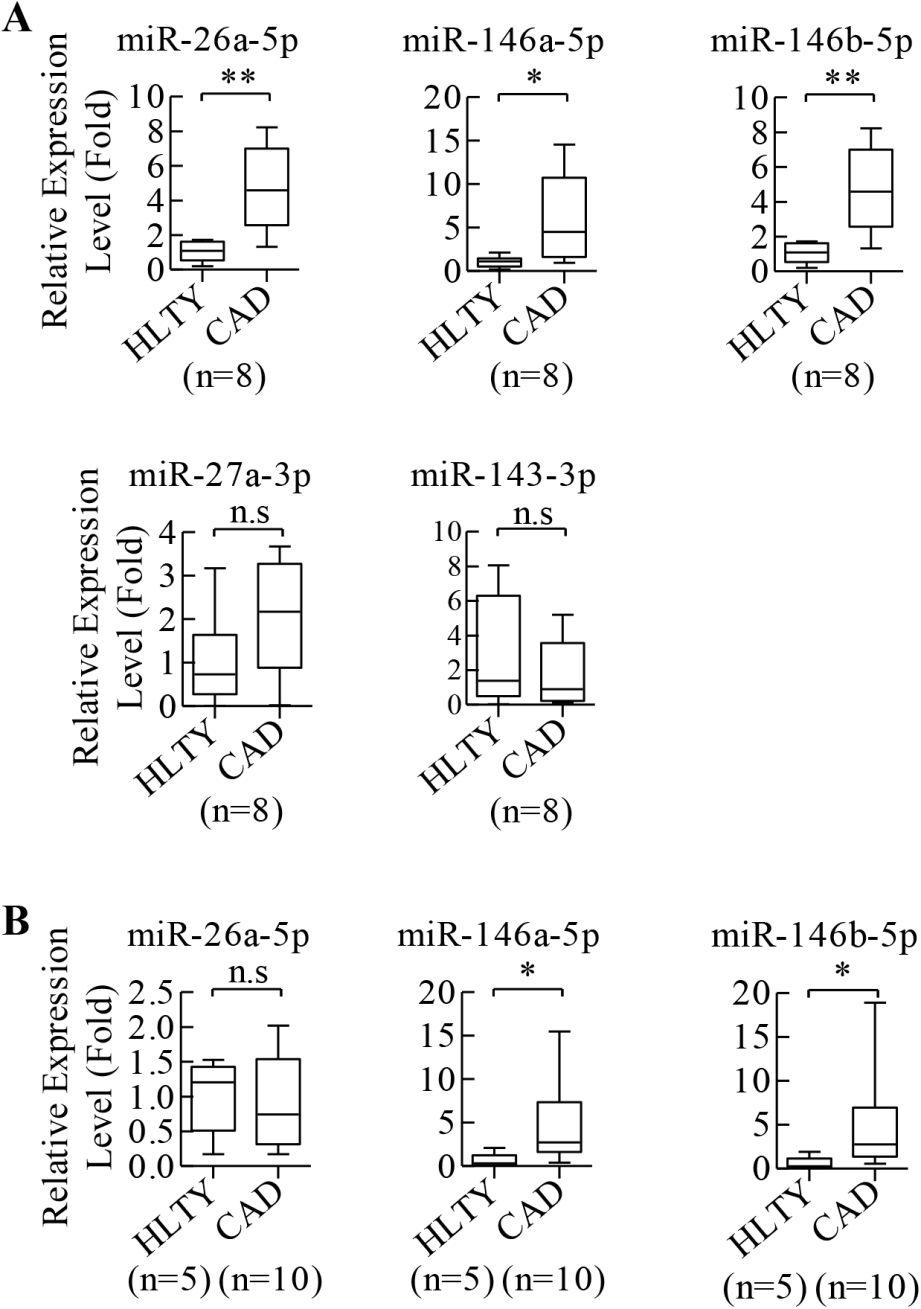
Reverse transcription-quantitative polymerase chain reaction (RT-qPCR) assays show higher miR-146a-5p and miR-146b-5p levels in plasma and ECFCs of CAD patients than in HLTY individuals. (A) Levels of plasma miR-26a-5p, miR-146a-5p and miR-146b-5p were increased in patients with CAD. (B) miR-146a-5p and miR-146b-5p were expressed at significantly higher level in CAD ECFCs. n.s: not significant, *p<0.05, **p<0.01 by *Student’s-t* test.

**Fig 5:**
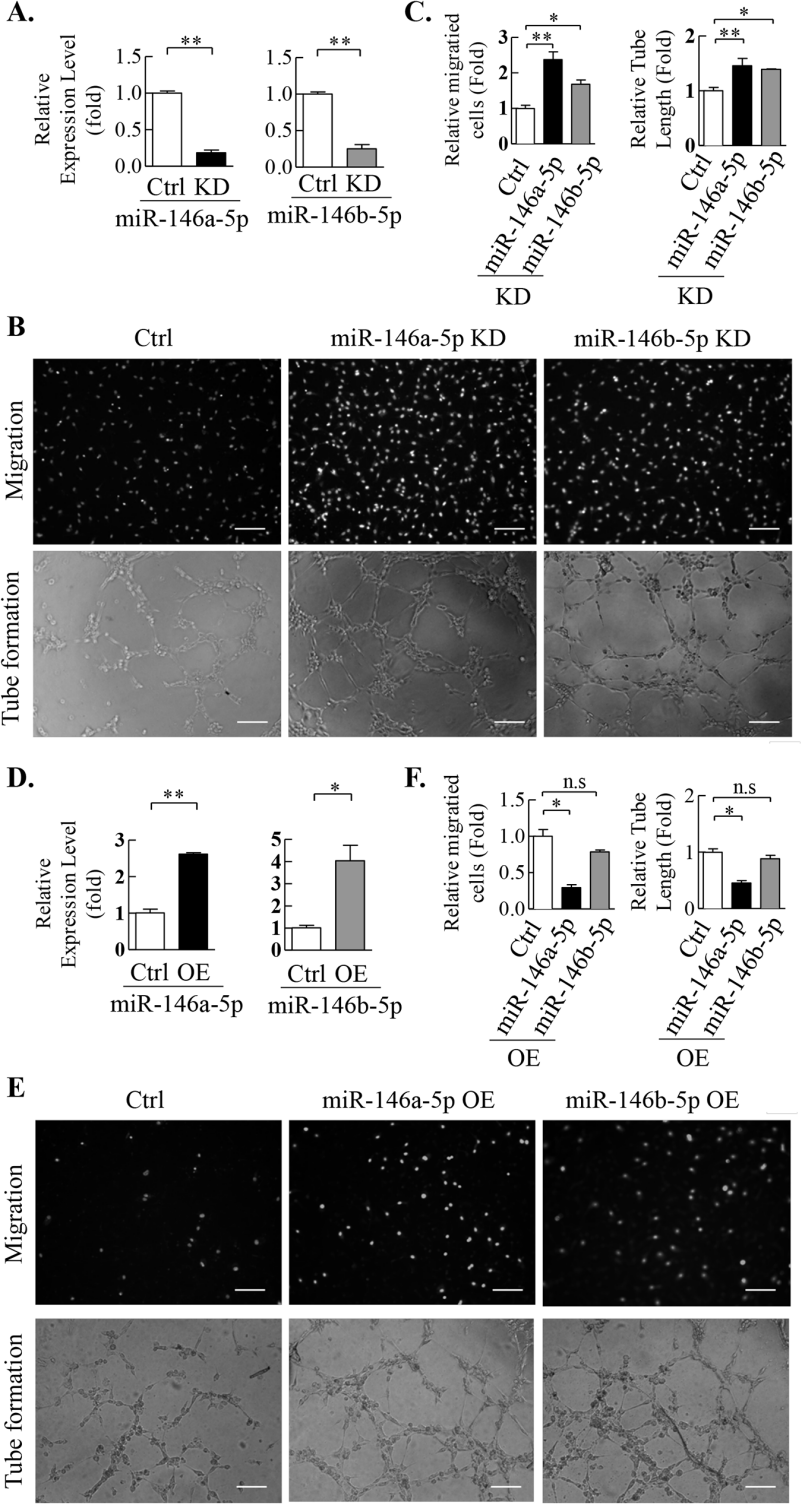
Functional characteristics of miR-146a-5p and miR-146b-5p as proangiogenic miRNAs. (A) Knockdown of miR-146a-5p and miR-146b-5p in CAD ECFCs by miRZip. Scramble oligonucleotide was used as control (Ctrl). (B) The proangiogenic effects of miR-146a-5p and miR-146b-5p. Transwell cell migration (upper panel) and tube formation (lower panel) assays were conducted on CAD ECFCs transduced with miRZip anti-miR-146a-5p (middle panel) and anti-miR-146b-5p (right panel). (C) Quantification data of (B). (D) Overexpression of miR-146a-5p and miR-146b-5p in HLTY ECFCs. (E) Transwell cell migration (upper panel) and tube formation (lower panel) assays were conducted on HLTY ECFCs transduced with lentivirus expressing miR-146a-5p (middle panel) and miR-146b-5p (right panel). (F) Quantification data of (E). n.s: not significant, *p<0.05, **p<0.01 by *Student’s-t* test.

Our *in silico* data was validated by detecting the levels of the five miRNAs in plasma and ECFCs from patients with CAD and HLTY control individuals. Among the five miRNAs examined, levels of circulating miR-26a-5p, miR-146a-5p and miR-146b-5p in plasma from CAD patients showed significant up-regulation compared to HLTY control (Fig 4A). Further exploration of their levels in ECFCs isolated from ten CAD patients and five HLTY control, only miR-146a-5p and miR-146b-5p were significantly more abundant in CAD cases (Fig 4B). No significant difference in miR-26a-5p level was observed between HLTY and CAD populations (Fig 4B). We then investigated the effect of miR-146a-5p and miR-146b-5p on ECFCs activities. Successful knockdown of endogenous miR-146a-5p and miR-146b-5p in CAD ECFCs by miRZip Anti-microRNA constructs were first confirmed by reverse transcription-quantitative polymerase chain reaction (RT-qPCR) (Fig 5A). Consistent with our hypothesis, angiogenic-related abilities, including migratory and microtubule formation activity, of CAD ECFCs were significantly increased in miR-146a-5p and miR-146b-5p knockdown cells (Fig 5B and 5C). More importantly, increasing miR-146a-5p or miR-146b-5p expression in HLTY ECFCs by transducing lentiviral vector expressing miR-146a-5p or miR-146b-5p (Fig 5D) resulted in a significant reduction in migration and microtubule formation activities of HLTY ECFCs (Fig 5E and 5F).

### miR-146a-5p and miR-146b-5p regulates ECFC function through suppressing a Rho-related GTP-binding protein RHOJ

**Fig 6:**
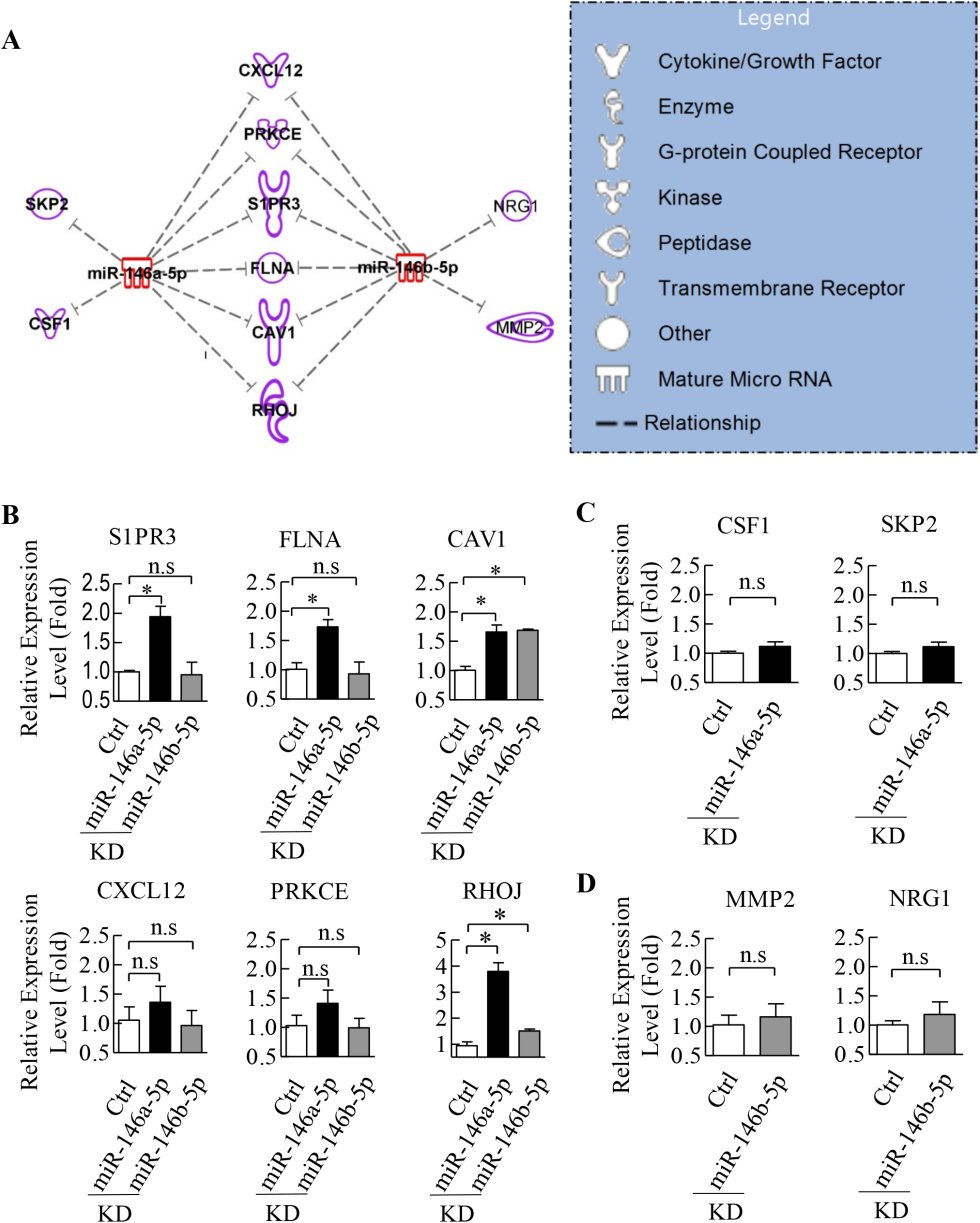
CAV1 and RHOJ are potential target genes of miR-146a-5p and miR-146b-5p. (A) Schematic presentation of the relationship between miR-146a-5p and miR-146b-5p with their predicted target genes. The function categories of listed genes are demonstrated in different shape according to the legends of right panel. Six genes were predicted to be negatively regulated by both miRNAs, while another 4 genes were predicted to be regulated by only one miRNA. (B) Knockdown of miR-146a-5p and miR-146b-5p in CAD ECFCs by miRZip significantly increased the expression of CAV1 and RHOJ. (C and D) Knockdown of miR-146a-5p (C) or miR-146b-5p (D) in CAD ECFCs by miRZip did not change the expression of its predicted target genes.

**Fig 7:**
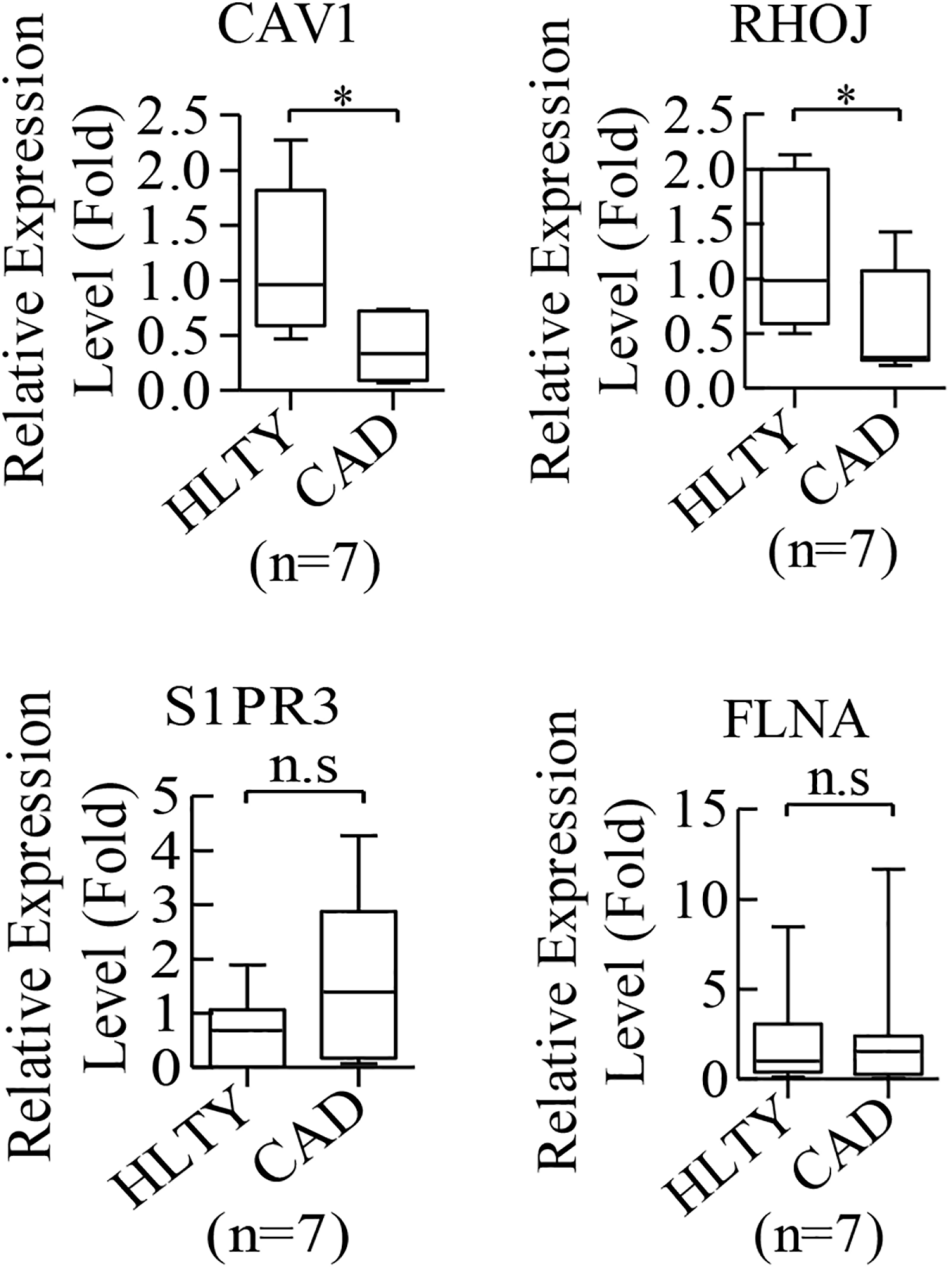
RT-qPCR assays show higher expression levels of CAV1 and RHOJ in CAD ECFCs than in HLTY ECFCs. Levels of CAV1 and RHOJ, but not S1PR3 and FLNA, were expressed at significantly higher level in CAD ECFCs. n.s: not significant, *p<0.05 by *Student’s-t* test.

To better understand the mechanism of how miR-146a-5p and miR-146b-5p regulate cell migration and microtubule formation, we attempted to identify potential novel downstream targets of miR-146a-5p and miR-146b-5p. TargetScan 7.0 bioinformatic algorithm was used to analyze 21 angiogenesis-related genes that were negatively correlated with miR-146a-5p and miR-146b-5p in CAD ECFCs in comparison with HLTY ECFCs (Fig 3C) to predict potential miR-146a-5p and miR-146b-5p targets. We identified a total of 10 target genes for miR-146a-5p and miR-146b-5p. Among them, 6 were targeted by both miR-146a-5p and miR-146b-5p, 2 were targeted by miR-146a-5p, and 2 were targeted by miR-146b-5p (Fig 6A). To further validate our *in silico* findings, we analyzed the expression levels of the 10 potential miRNA target genes in control and miR-146a-5p and miR-146b-5p knockdown CAD ECFCs using RT-qPCR. The expression of CAV1 and RHOJ that were identified as target genes of both miR-146a-5p and miR-146b-5p were significantly increased in both miR-146a-5p and miR-146b-5p knockdown CAD ECFCs (Fig 6B). Though S1PR3 and FLNA were also identified as target genes of both miR-146a-5p and miR-146b-5p, their RNA levels were found to be increased in CAD ECFCs only when miR-146a-5p was knockdown (Fig 6B). The expression level of CSF1 and SKP2 showed no significant change upon miR-146a-5p knockdown in CAD ECFC (Fig 6C). Moreover, levels of MMP2 and NRG1 were not increased upon miR-146b-5p knockdown (Fig 6D). These results suggest that CAV1, RHOJ, S1PR3 and FLNA are potential angiogenesis-related genes targeted by miR-146a-5p and miR-146b-5p in CAD ECFCs. To further confirm this, we analyzed the expression levels of CAV1, RHOJ, S1PR3 and FLNA in both HLTY and CAD ECFCs by RT-qPCR and showed lower expression levels of both CAV1 and RHOJ in CAD ECFCs when compared with HLTY ECFCs (Fig 7). Together, all these data indicate that CAV1 and RHOJ are novel targets of miR-146a-5p and miR-146b-5p in CAD ECFC.

**Fig 8:**
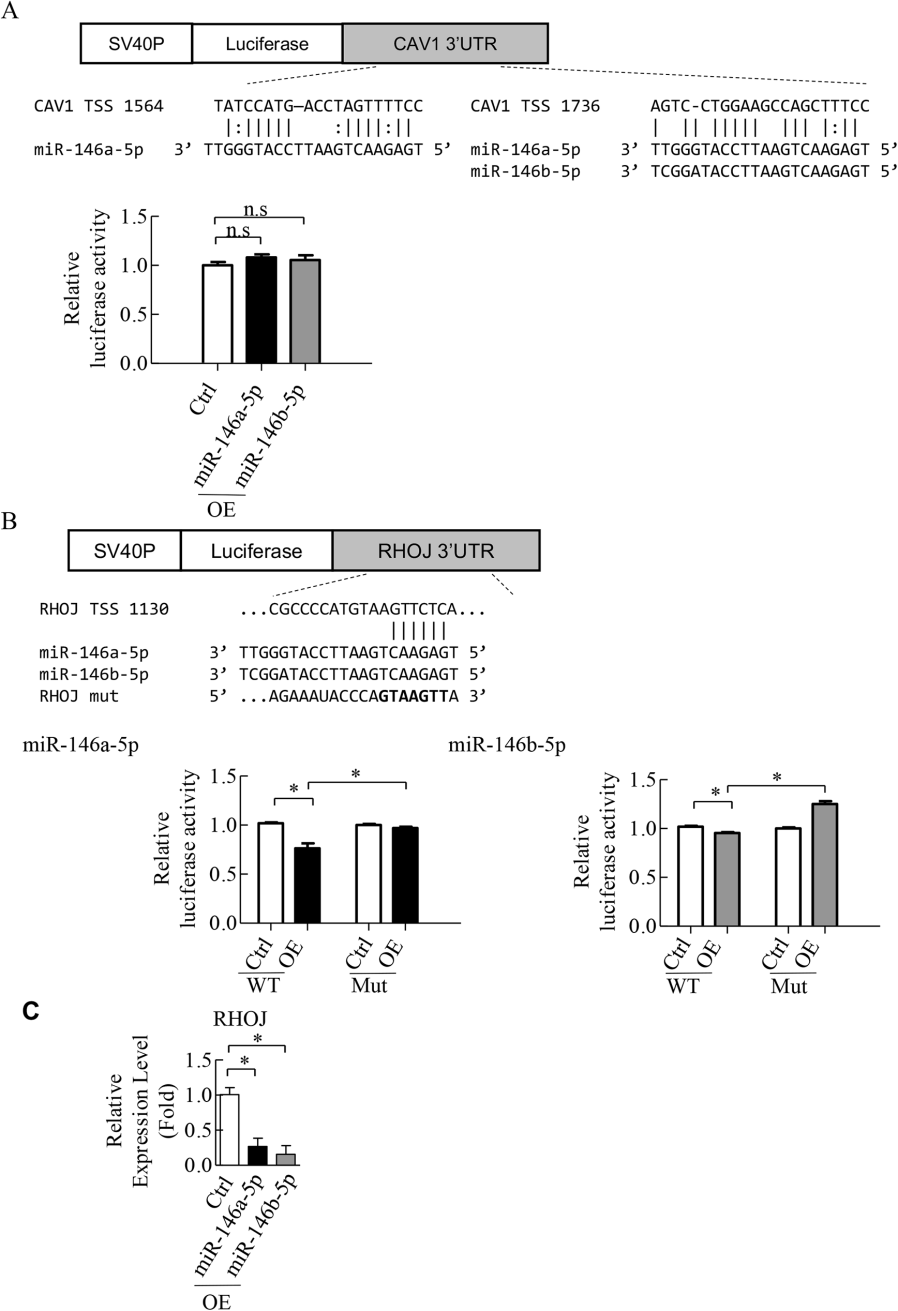
miR-146a-5p and miR-146b-5p directly target RHOJ. (A) Structure of the luciferase reporter construct and the predicted miR-146a-5p and miR-146b-5p binding site(s) on 3’UTR of CAV1 (upper panel). Luciferase activities of 293T cells co-transfected with reporter plasmid and control (Ctrl), miR-146a-5p or miR-146b-5p precursor (lower panel). (B) Structure of the luciferase reporter construct and the predicted miR-146a-5p and miR-146b-5p binding site(s) on 3’UTR of RHOJ (upper panel). The luciferase reporter plasmids containing either miR-146a-5p (lower left panel) and miR-146b-5p (lower right panel) binding site (WT) or miRNA-binding deficient mutant (Mut) were co-transfected with miR-146a-5p (lower left panel) and miR-146b-5p (lower right panel). Luciferase activity of individual miRNAs co-transfected with either WT or Mut reporter plasmids (lower panel). (C) The expression levels of RHOJ in HLTY ECFCs overexpressing miR-146a-5p and miR-146b-5p were detected by RT-qPCR.

**Fig 9:**
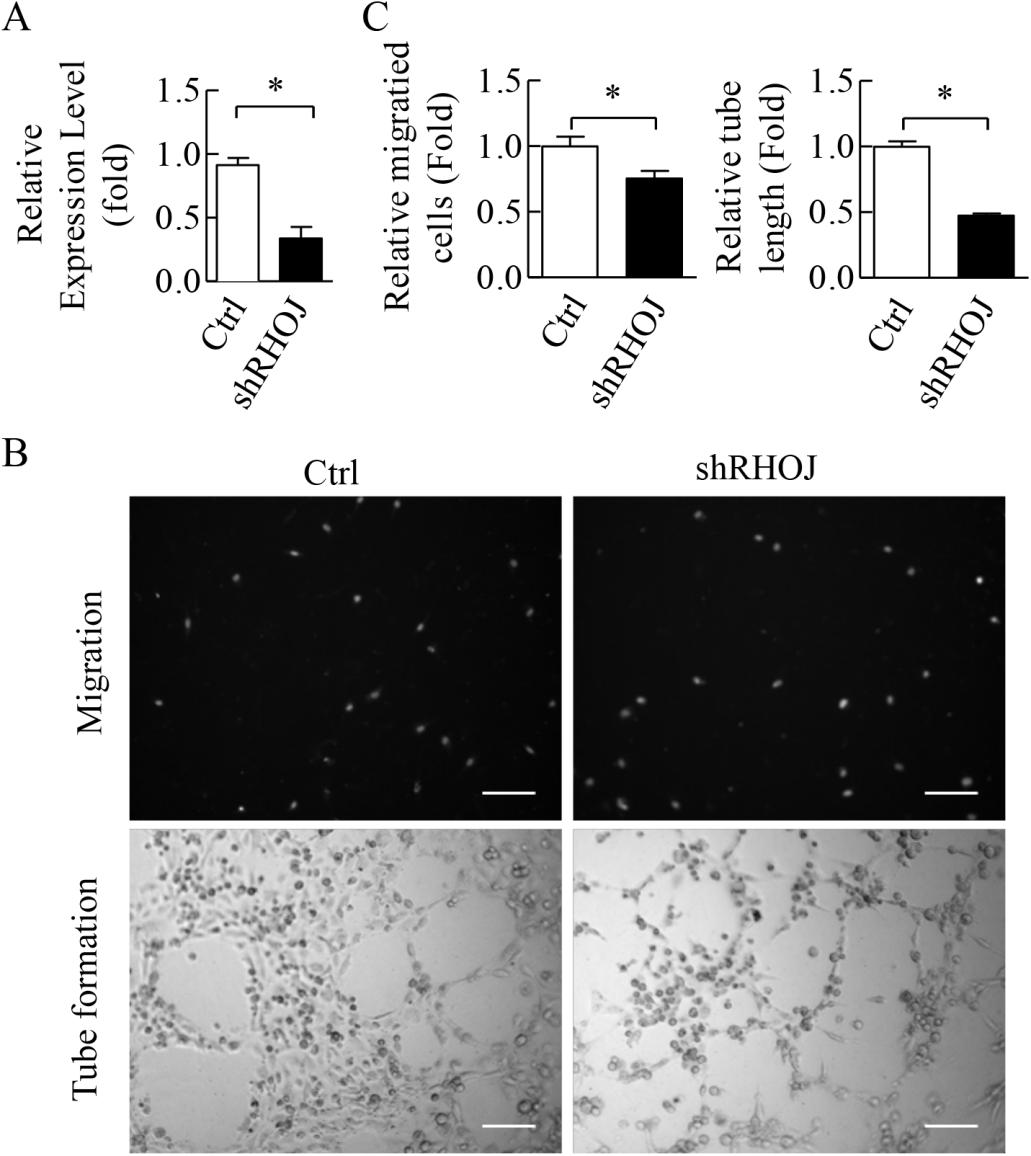
Functional characteristics of RHOJ as a proangiogenic gene. (A) Knockdown of RHOJ in HLTY ECFCs by shRHOJ. Scramble oligonucleotide was used as a negative control (Ctrl). (B) The proangiogenic effects of RHOJ. Transwell cell migration (upper panel) and tube formation (lower panel) assays were conducted on HLTY ECFCs transduced with lentivirus overexpressing shRHOJ (right panel). (C) Quantification data of (B). *p<0.05 by *Student’s-t* test.

The direct repression of CAV1 and RHOJ by miR-146a-5p and miR-146b-5p was explored using luciferase reporter assay. TargetScan 7.0 was utilized to pinpoint the two predicted binding sites for miR-146a-5p (TSS 1564 and TSS 1736), one binding site for miR-146b-5p (TSS 1736) on the 3’-untranslated region (3’-UTR) of CAV1 (Fig 8A, upper panel) and one binding site for both miR-146a-5p and miR-146b-5p on the 3’UTR of RHOJ (TSS 1130) (Fig 8B, upper panel). The 3’-UTR of CAV1 and RHOJ containing seed sequence of miR-146a-5p and miR-146b-5p were inserted downstream of luciferase cDNA. We found that miR-146a-5p and miR-146b-5p were able to repress luciferase expression when the construct contained the 3’-UTR of RHOJ (Fig 8B) but not of CAV1 (Fig 8A). This repression of luciferase activity was reversed when the binding site for miR-146a-5p and miR-146b-5p in RHOJ 3’-UTR was mutated (Fig 8B). Furthermore, we confirmed by RT-qPCR that the expression levels of RHOJ were significantly decreased when miR-146a-5p and miR-146b-5p were overexpressed in HLTY ECFCs (Fig 8C). RHOJ is a Rho GTPase mainly expressed in endothelial cells and has been shown to regulate endothelial motility and tubulogenesis [44, 45]. To further evaluate the effect of RHOJ in ECFCs, we knocked down RHOJ in HLTY ECFCs. Successful knockdown of endogenous RHOJ in HLTY ECFCs by lentiviral vector expressing shRHOJ was confirmed by RT-qPCR (Fig 9A). Consistently, knockdown of RHOJ significantly inhibit migration and microtubule formation activities of HLTY ECFCs (Fig 9B and 9C). These data together indicate that RHOJ is a novel direct target of miR-146a-5p and miR-146b-5p, and it can cause dysfunction of angiogenic-related abilities of ECFCs.

## Discussion

Endothelial damage is considered as the first step of the cascade in arterial pathogenesis and therefore has long been believed to play a pivotal role in the development of CAD. Repair of defective endothelium at adult stage is not solely a result of proliferation of local endothelial cell, but also depends largely on differentiation of blood mononuclear cells derived ECFCs into endothelial cells [46]. Reduced ECFC-mediated vascular repair functionality is believed to contribute to increase the risk of CAD. In this report, we found that CAD ECFCs may also exploit the secretory pathway in an opposite manner as a means of reducing angiogenesis- and vasculogenesis-related activities of HLTY ECFCs. The CAD ECFCs secreted factors, which can travel as free form (Fig 1B) or in exosomes (Fig 1C), were sufficient for, as well as being critical to, suppressing migration and tube formation activity of HLTY ECFCs. This finding suggests that diseased ECFCs in CAD may not only reduce blood vessel repair by loss of its own function but also by secreting inhibitory factors to inhibit the repair of endothelium and by dysregulating the functions of HLTY ECFCs. Identifying and blocking this negative-feedback loop may provide a new strategy to slow down the progression of CAD and other EPC-related diseases, such as diabetes mellitus-related arterial disorder.

The miRNAs expression profiles of HLTY and CAD ECFCs have been extensively studied. There is emerging evidence showing many anti-angiogenic miRNAs, which target angiogenic genes such as VEGF, are up-regulated in CAD ECFCs and consequently suppresses ECFC activities [33–35]. It is conceivable that angiogenic cytokines are some of the crucial targets of the miRNAs found up-regulated in CAD ECFCs. However, the role of miRNAs in targeting other angiogenic-related genes in CAD ECFC was largely unknown. In this study, by combining transcriptome-based profiling of mRNA and miRNA expression with bioinformatics analysis and prediction, we identified the miR-146a-5p/RHOJ and miR-146b-5p/RHOJ axis as potential novel mechanism underlying CAD pathogenesis and prognostic biomarker for CAD patients. miR-146a-5p has been initially identified as a cancer-related miRNA. Some studies have implicated it as an oncogenic miRNA [47, 48], but others suggested a carcinostatic role [49–51]. Some recent reports indicate the function of miR-146a-5p in endothelial cells. However, most studies focus on its inflammatory roles [52, 53]. Though, one recent report showed lower level of miR-146a-5p and higher levels of VEGF-A in endometriotic lesions suggesting a potential role of miR-146a-5p in targeting proangiogenic factors in angiogenesis [54], little is known about the expression and exact function of miR-146a-5p in ECFCs. Similarly, miR-146a-5p was found to participate in inflammatory functions of endothelial cells [55]. Significant up-regulation of miR-146b-5p was identified in pulmonary artery remodeling indicating its role in angiogenesis [56]. However, there are no evidence to show a link between miR-146b-5p and ECFCs. Together, these results indicate the potential of miR-146a-5p and miR-146b-5p in regulating angiogenesis, but little is known about the role of miR-146a-5p and miR-146b-5p in regulating ECFCs activities and vascular repair. Here, we showed that miR-146a-5p and miR-146b-5p were up-regulated in both plasma (Fig 4A) and ECFCs (Fig 4B) of CAD patients. Knockdown of miR-146a-5p and miR-146b-5p increased the migration and tube formation activity of CAD ECFCs (Fig 5A). Consistently, overexpression of miR-146a-5p and miR-146b-5p significantly inhibited the migration and tube formation activity of HLTY ECFCs (Fig 5B). Since the activities of ECFCs have been shown to correlate with the integrity of endothelium and severity of CAD, our finding may have prognostic implication of combined liquid biopsy biomarker in CAD and clinical implications for development of CAD treatment involving inhibition of multiple miRNAs.

miRNAs play a key regulatory role in gene expression at the posttranscriptional level. It is well recognized that miRNA represses gene expression at both the level of mRNA stability by inducing mRNA degradation and the level of translation inhibition by binding to 3′UTR of the target mRNAs [57]. Here, we focus on miRNA mediated RNA degradation and performed cDNA microarray analysis to identify genes potentially targeted and degraded by miR-146a-5p and miR-146b-5p. Though several miR-146a-5p [58] and miR-146b-5p [59] targeting genes were identified, these genes were mainly related to cancer. To elucidate the role of miR-146a-5p and miR-146b-5p in angiogenesis, we performed an integrative bioinformatics analysis of transcriptome and miRNome (Fig 3) and revealed the regulatory network involved in angiogenesis (Fig 6A). Among the genes identified, S1PR3 and FLNA were increased in CAD ECFCs when miR-146a-5p was knockdown and RHOJ and CAV1 were significantly up-regulated in both miR-146a-5p and miR-146b-5p knockdown CAD ECFCs (Fig 6B). Although all four genes that we validated were up-regulated in knockdown of miR-146a-5p or/and miR-146b-5p, only RHOJ and CAV1 were consistently much lower in the ECFCs of CAD patients when compared with healthy control (Fig 7). These data indicate that multiple miRNAs may be required for dysregulation of angiogenic genes in CAD. Reporter assays confirmed the direct binding and repression of both miR-146a-5p and miR-146b-5p to the 3’-UTR of VEGF mRNA (Fig 8B). Consistently, overexpression of miR-146a-5p and miR-146b-5p significantly decreased the expression level of RHOJ in HLTY ECFCs (Fig 8C). RHOJ belongs to the Rho GTPase subfamily expressed mainly in endothelial and tumor cells (reviewed in [60]). Functionally, RHOJ was shown to regulate endothelial motility and tubulogenesis *in vitro* [44, 61] and vascularization *in vivo* [61, 62]. However, there is no report showing the role of RHOJ in ECFCs. In this study, we showed for the first time that RHOJ also plays an important role in maintaining the normal function of HLTY ECFCs (Fig 9B and 9C). Together, our data indicate that RHOJ is a novel direct target gene of miR-146a-5p and miR-146b-5p in ECFCs and can cause dysfunction of angiogenic-related abilities of ECFCs. The role of RHOJ in endothelial cells suggests that miRNAs secreted by CAD ECFCs may not only be taken up by HLTY ECFCs but also by endothelial cells and consequently disrupt the normal function of endothelial cells.

**Fig 10:**
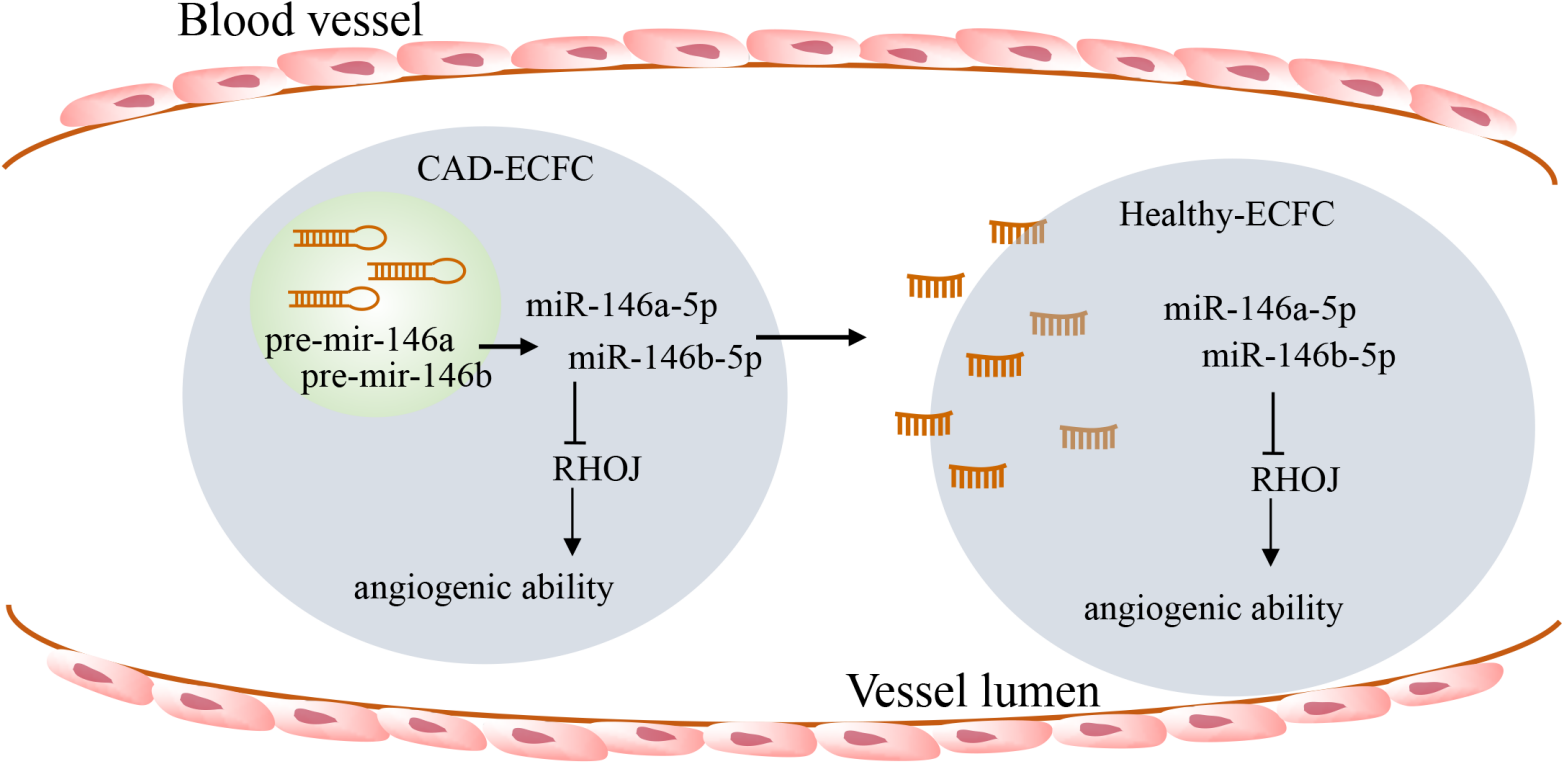
A schematic model of CAD ECFCs secreted miR-146a-5p and miR-146b-5p in plasma and uptake by HLTY ECFCs that consequently dysregulated RHOJ expression and functionality in HLTY ECFCs making them more like CAD ECFCs.

In summary, our study has revealed miR-146a-5p and miR-146b-5p as new potential angiogenesis regulatory miRNAs that are up-regulated and potentially secreted by CAD ECFCs. Here, we provide indirect evidence showing that circulating miR-146a-5p and miR-146b-5p, including free and exosome-associated forms, may be taken up by HLTY ECFCs and resulting in amplification of diseased ECFCs (Fig 10). Our results also reveal the existence of miR-146a-5p/RHOJ and miR-146b-5p/RHOJ axis as a novel pathogenesis pathway that regulates ECFC function in patients with CAD. Identifying and blocking this secretory miRNAs-mediated negative-feedback loop may provide a novel strategy for therapeutic application in CAD and other EPC-related diseases.

## Supporting information

S1 Fig Characterization of HLTY and CAD ECFCs. Expression of indicated molecules in HLTY and CAD ECFCs were stained and analyzed by flow cytometer.
(TIF)

S2 Fig Heat map of miRNAs that are significantly higher expressed in plasma and EPCs of CAD patients when compared with HLTY individuals. (A) Heat map of 82 miRNAs significantly higher expressed in CAD plasma (RPM > 5 and > 2-fold change). (B) Heat map of 83 miRNAs significantly highly expressed in in CAD ECFCs (RPM > 5 and > 3-fold change).
(TIF)
