# Supplementary figures and images for: Dysregulation of endothelial colony-forming cell function by a negative feedback loop of circulating miR-146a and -146b in cardiovascular disease patients
Source: PLoS One. 2017 Jul 20;12(7):e0181562. doi: 10.1371/journal.pone.0181562 (PMC5519171; doi:10.1371/journal.pone.0181562)

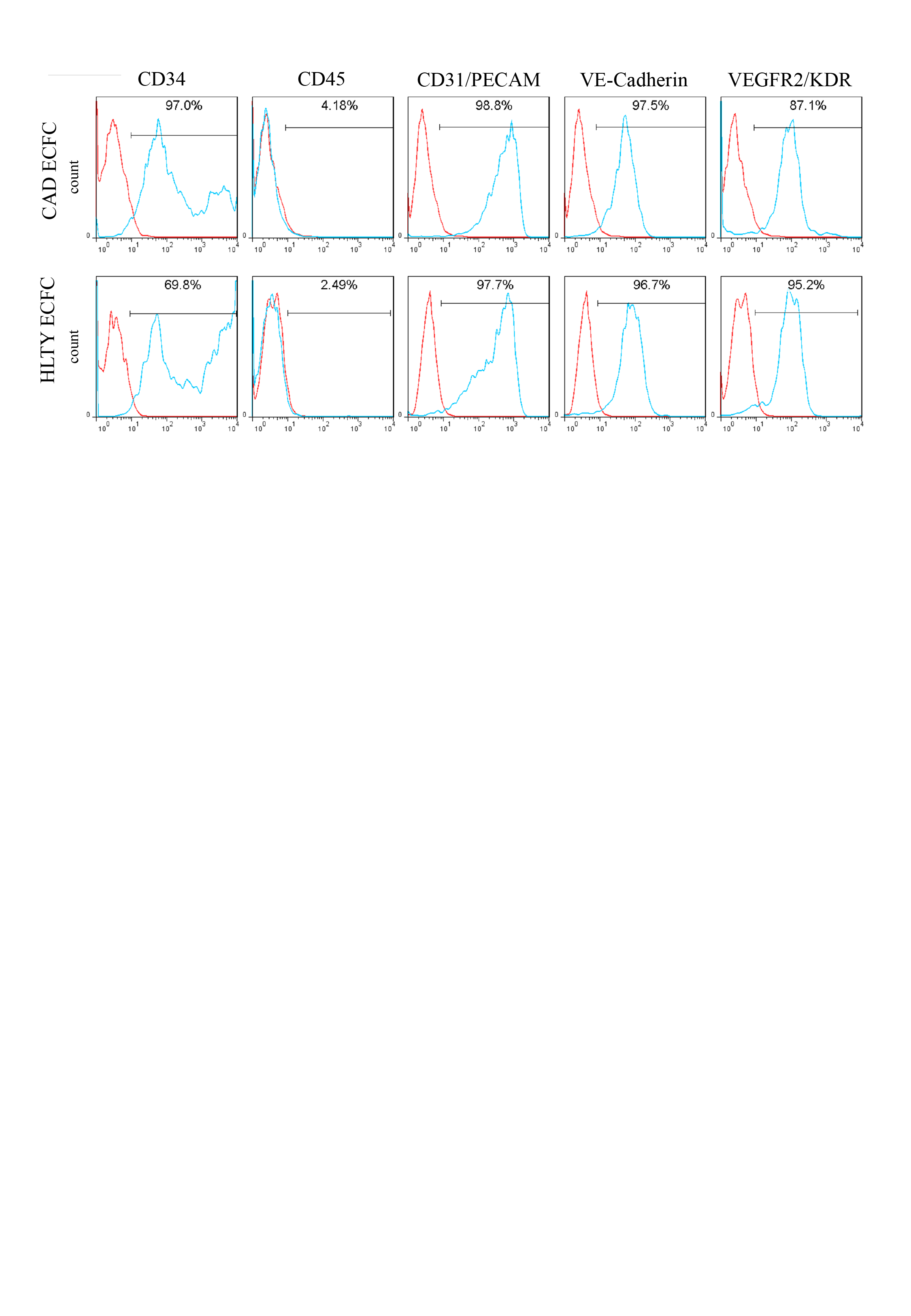

Supplement: S1 Fig — Expression of indicated molecules in HLTY and CAD ECFCs were stained and analyzed by flow cytometer. (TIF) [file pone.0181562.s001.tif]

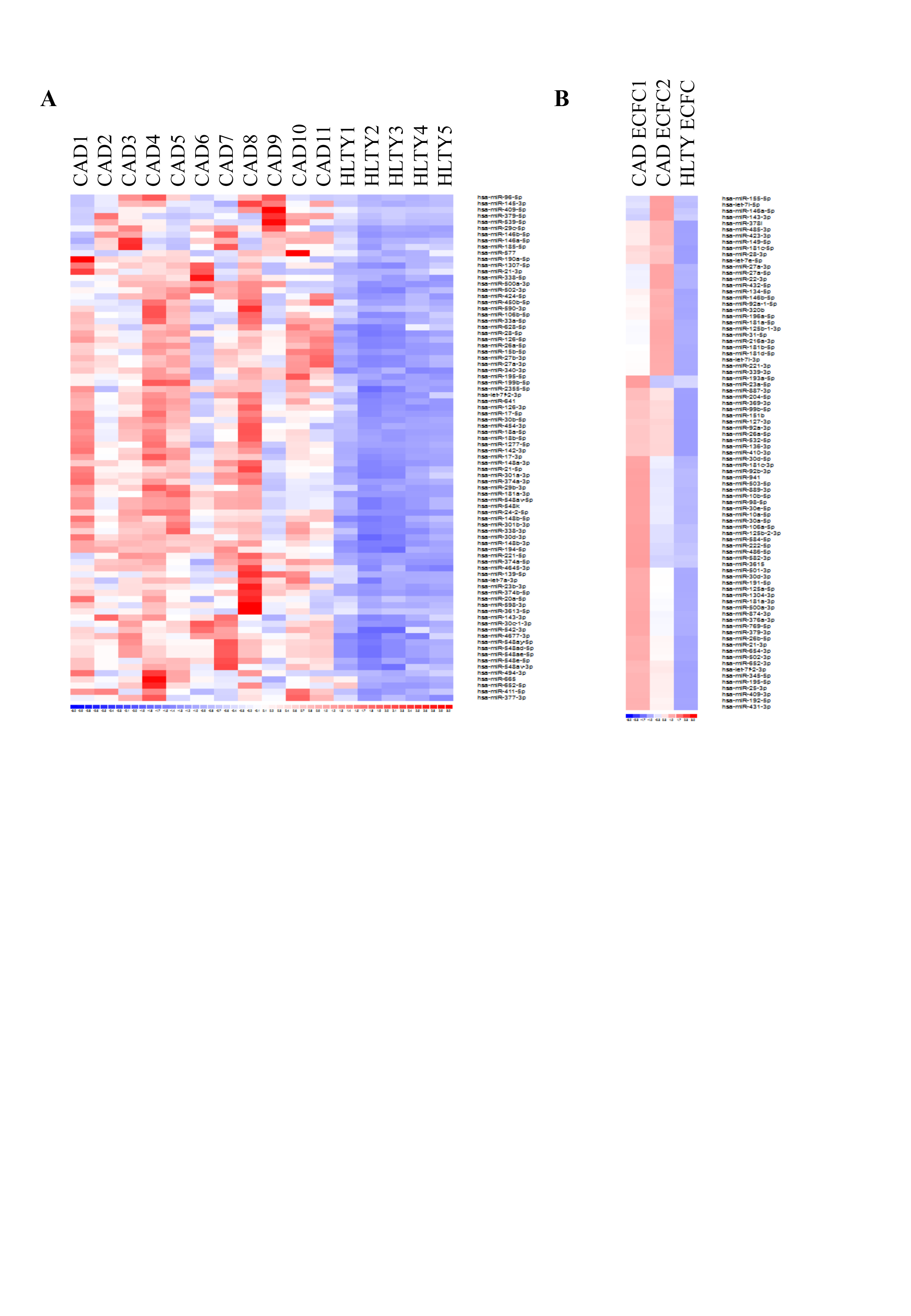

Supplement: S2 Fig — (A) Heat map of 82 miRNAs significantly higher expressed in CAD plasma (RPM > 5 and > 2-fold change). (B) Heat map of 83 miRNAs significantly highly expressed in in CAD ECFCs (RPM > 5 and > 3-fold change). (TIF) [file pone.0181562.s002.tif]
